# Phase II trial of natalizumab for the treatment of anti-Hu associated paraneoplastic neurological syndromes

**DOI:** 10.1093/noajnl/vdab145

**Published:** 2021-09-28

**Authors:** Anna E M Bastiaansen, Adriaan H C de Jongste, Marienke A A M de Bruijn, Yvette S Crijnen, Marco W J Schreurs, Marcel M Verbeek, Daphne W Dumoulin, Walter Taal, Maarten J Titulaer, Peter A E Sillevis Smitt

**Affiliations:** 1 Department of Neurology, Erasmus MC University Medical Center, Rotterdam, The Netherlands; 2 Department of Neurology, Elisabeth Tweesteden Medical Center, Tilburg, The Netherlands; 3 Department of Immunology, Erasmus MC University Medical Center, Rotterdam, The Netherlands; 4 Department of Neurology and Laboratory Medicine, Donders Institute for Brain Cognition and Behavior, Radboud University Medical Center, Nijmegen, The Netherlands; 5 Department of Pulmonary Medicine, Erasmus MC Cancer Institute, Rotterdam, The Netherlands

**Keywords:** antibodies, anti-Hu, natalizumab, paraneoplastic neurological syndromes, treatment

## Abstract

**Background:**

Paraneoplastic neurological syndromes with anti-Hu antibodies (Hu-PNS) have a very poor prognosis: more than half of the patients become bedridden and median survival is less than 12 months. Several lines of evidence suggest a pathogenic T cell-mediated immune response. Therefore, we conducted a prospective open-label phase II trial with natalizumab.

**Methods:**

Twenty Hu-PNS patients with progressive disease were treated with a maximum of three monthly natalizumab cycles (300 mg). The primary outcome measure was functional improvement, this was defined as at least one point decrease in modified Rankin Scale (mRS) score at the last treatment visit. In addition, treatment response was assessed wherein a mRS score ≤3 after treatment was defined as treatment responsive.

**Results:**

The median age at onset was 67.8 years (SD 8.4) with a female predominance (*n* = 17, 85%). The median time from symptom onset to Hu-PNS diagnosis was 5 months (IQR 2–11). Most patients had subacute sensory neuronopathy (*n* = 15, 75%), with a median mRS of 4 at baseline. Thirteen patients had a tumor, all small cell lung cancer. After natalizumab treatment, two patients (10%) showed functional improvement. Of the remaining patients, 60% had a stable functional outcome, while 30% showed further deterioration. Treatment response was classified as positive in nine patients (45%).

**Conclusions:**

Natalizumab may ameliorate the disease course in Hu-PNS, but no superior effects above other reported immunosuppressive and immunomodulatory were observed. More effective treatment modalities are highly needed.

**Trial registration:**

https://www.clinicaltrialsregister.eu/ctr-search/trial/2014-000675-13/NL

**Key Points**

- Hu-PNS has a very poor prognosis with functional improvement in <10% of patients.
- Natalizumab showed no superior efficacy above other previously studied immunosuppressive and immunomodulatory therapies.
- More effective treatments are a highly unmet medical need.

**Importance of the Study**

Paraneoplastic neurological syndromes with anti-Hu antibodies (Hu-PNS) are associated with malignancies, predominantly small cell lung cancer. It is important to search for an underlying malignancy and start antitumor treatment as early as possible. Previous trials with immunosuppressive or immunomodulatory treatment in Hu-PNS obtained functional improvement in a minority of patients (around 10%). Several lines of evidence suggest that neuronal damage in Hu-PNS is caused by a pathogenic T cell-mediated immune response. Therefore, we conducted a phase II trial of natalizumab, a monoclonal antibody that strongly inhibits the migration of activated T lymphocytes into the central nervous system and that inhibits activated T cells. This study shows that natalizumab is probably not more effective in treatment of Hu-PNS than previously studied immunosuppressive and immunomodulatory therapies.

Paraneoplastic neurological syndromes (PNS) are rare immune-mediated neurological disorders associated with malignancies. PNS with anti-Hu antibodies (Hu-PNS) are the most frequent among the PNS associated with well-characterized onconeural antibodies. The underlying tumor in Hu-PNS is most often small cell lung cancer (SCLC). Hu-PNS is a severe disease progressing rapidly over weeks to months and has a poor prognosis: more than half of the patients become bed or wheelchair-bound, only 5–7% of patients improve and the median survival is less than one year.^1,2^ At the time of neurological presentation, the patient is not aware of the cancer in over 70%, delaying the diagnosis of Hu-PNS.^1–4^ It is thought that the expression of Hu antigens by the tumor provokes an autoimmune response not only directed against the tumor but also against nervous tissues.^5^ Although the anti-Hu antibodies (Hu-Ab) are present in high titers in serum and CSF, neuronal destruction in Hu-PNS is more likely caused by T cells than by Hu-Ab. Hu proteins are intracellular proteins that can not be reached by antibodies and many animal models failed to demonstrate Hu-Ab induced disease. Furthermore, autopsy studies consistently showed T cell infiltrates with cytotoxic T cells frequently surrounding neurons with associated neuronal loss.^6–8^

Natalizumab strongly inhibits the migration of activated T lymphocytes into the central nervous system (CNS) and is approved for the treatment of relapsing-remitting multiple sclerosis.^9^ In addition, it may contribute to reduced activation of T cells already present in the CNS, leading to increased apoptosis of pathogenic T cells and lowering damage done to the nervous system.^10,11^

We conducted a prospective open-label single-arm trial to evaluate the efficacy of off-label use of natalizumab in patients with progressive Hu-PNS. We monitored function and neurological impairment using well-defined clinical scales, as well as toxicity.

## Patients and Methods

### Patients

At the Erasmus University Medical Center, the Departments of Neurology and Medical Immunology are the national referral centers for anti-neuronal antibody testing, diagnosis, and treatment, accredited as European Reference Network site (ERN-RITA). Between March 2016 and June 2020, 80 patients were identified with increased serum titers of Hu-Ab (titer ≥400 by indirect immunofluorescence on monkey cerebellum, and confirmed by Euroimmun [Lübeck, Germany] and RAVO Diagnostika [Freiburg, Germany] blots). Inclusion criteria comprised a paraneoplastic neurological syndrome associated with increased Hu-Ab titer, progression of neurological symptoms over the last 4 weeks, a modified Rankin Scale (mRS)^12^ score ≥2, age ≥18 years, and an absolute CD4+ cell count ≥0.4 x 10^9^ cells/liter. Exclusion criteria were unwillingness to undergo a lumbar puncture, known hypersensitivity to natalizumab or one of the additives, progressive multifocal leukoencephalopathy (PML), immune-compromized patients (patients using immunosuppressive medications other than a short course (<2 weeks) of steroids), liver and renal failure, active infections, pregnancy, a history of active melanoma in the past 5 years, and T cell lymphoma or primary CNS lymphoma.

**Figure 1.**
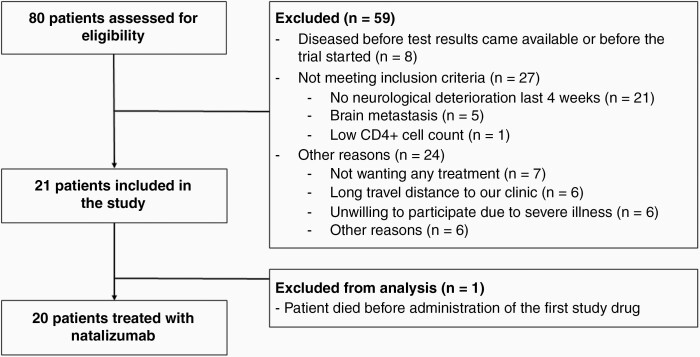
Patient inclusion. In total, 80 patient with a high serum titer of Hu-Ab (≥400) were identified between March 2016 and June 2020. Twenty-one patients were included in this study, one of whom died before administration of study medication and was excluded from analysis. In total, 20 patients were treated with natalizumab and included for analysis.

Of the 80 identified patients, 59 were excluded due to factors depicted in Figure 1. The remaining 21 patients were included in the trial and gave written informed consent. One of the patients died unexpectedly before administration of the first study medication and was excluded from the analysis.

We performed immunohistochemistry (IHC) to detect additional antibodies against extracellular neuronal proteins, on all sera and CSF samples of the included patients.^13,14^ When positive, confirmatory laboratory analyses were performed using validated commercial cell-based assays (CBA) or live hippocampal neurons as described before.^13,14^

### Study Design and Treatment

We performed an open-label single-arm, single-center phase II study. The 20 treated patients were treated with a maximum of three monthly cycles of natalizumab (intravenous infusions of 300 mg). Patients visited our clinic at least for every treatment cycle, four weeks after the third infusion and the last study visit occurred twenty weeks after the start of the trial (12 weeks after the last natalizumab cycle). Each study visit, patients were subjected to clinical evaluation, toxicity monitoring (according to the Common Terminology Criteria for Adverse Events (CTCAE)), and laboratory analysis. Natalizumab was used as monotherapy and concomitant immunotherapy was not allowed. Treatment of an underlying malignancy, including chemotherapy, was allowed (PD-(L)1 checkpoint inhibition was not standard care for SCLC in the Netherlands). The study drug was discontinued when the mRS score increased ≥2 points or in case of intolerable toxicity. Use of natalizumab in multiple sclerosis has been associated with an increased risk of progressive multifocal leukoencephalopathy (PML), an opportunistic infection caused by John Cunnningham virus, which may be fatal or result in severe disability.^9,15^ However, the intention to treat patients with only 3 cycles of natalizumab (12 weeks) renders the occurrence of PML very unlikely as most cases have occurred after >2 years of treatment. A data safety monitoring board was assigned to assess toxicity and review all serious adverse events.

This study was approved by the Institutional Review Board of Erasmus MC (MEC-2015-607). Guidelines for neuro-oncology: Standards for investigational studies were followed (GNOSIS).^16^

### Outcome

The primary outcome measure of this study was functional improvement, defined as at least one point decrease in mRS score after 12 weeks compared to baseline mRS score. We used a standardized mRS algorithm to achieve consistent scores.^17^ In addition, we performed explorative analyses using the criteria for treatment response by Keime-Guibert et al.^18^ in our cohort, and for comparison with previous studies. A positive treatment response was defined by these authors as improvement or stabilization in patients with an mRS score ≤3, and an improvement from mRS ≥4 to mRS ≤3. For both outcome scores, we additionally analyzed mRS scores at 20 weeks compared to baseline.

The first secondary outcome measure was neurological improvement, assessed using the Edinburgh Functional Impairment Tests (EFIT).^19^ The EFIT integrates upper and lower limb function, memory, and a rating scale for dysphasia. Neurological improvement was defined as one-point increase in overall EFIT score. Secondly, limitations in daily living activities were evaluated using the Barthel index (BI).^20^

The prospective open-label sirolimus trial, conducted in 17 patients with Hu-PNS in our institution from 2008 to 2012, was used as a historical control group.^21^

### Statistical Analysis

Based on previous studies, the chance of improvement ≥1 point in mRS score in historical Hu-PNS controls was put at 10%.^1,2,18,21,22^ We designed the study to detect improvement in 35% of the patients following natalizumab treatment. To achieve power of 80% with two sided α = 0.05, we calculated a sample size of 18 patients. To allow for 10% drop-outs we intended to include 20 patients. Statistical analysis was performed using SPSS 25.0 (IBM, New York, NY) for Windows, as well as Prism 8.4.3 (GraphPad Prism Software Inc., San Diego, CA). All p-values were two-sided and were considered statistically significant when below 0.05. Patient-specific baseline characteristics were evaluated using standard descriptive features: mean with standard deviation, median with interquartile range (IQR) and range for continuous variables, and frequency (proportions) for categorical variables. For group comparisons, encompassing categorical data, we used the Pearson Chi-Square test or the Fisher-Exact test if appropriate. Continuous data were analyzed using the Student’s t-test or the Mann-Whitney U test in case of skewed distribution. Wilcoxon matched-pairs test was used to compare Hu-Ab titers in serum and CSF at baseline and after treatment (12 weeks after start trial).

## Results

### Patients, Treatment, and Toxicity

In total, 20 patients were treated with natalizumab (Table 1, and baseline cohort characteristics in Supplementary Table 1). The median age at onset was 67.8 (SD ±8.4) and there was a female predominance (*n* = 17, 85%). Diagnosis of Hu-PNS took a median of 5 months (IQR 2–11) from symptom onset. In most patients, the dominant clinical syndrome was subacute sensory neuronopathy (SSN, *n* = 6) or SSN combined with other symptoms (total *n* = 15, 75%). Their median mRS at baseline was 4 (range 2–5).

**Table 1.** Patient and Tumor Characteristics

No.	Age/ Sex	PNS—Clinical Phenotype	Onset to Diagnosis Hu-PNS (months)	Tumor	Onset to Diagnosis Tumor (months)	Tumor Stage	Tumor Treatment	Tumor Response
1	64/F	SSN, MN, PLE	5	SCLC	5	LD	Chemo, RT	CR
2	57/F	SSN	62	SCLC	6	ED	Chemo, RT ^a^	CR
3	66/F	SSN	3	SCLC	5	LD	Chemo, RT	CR
4	53/F	SSN, AN	2	SCLC	3	LD	Chemo, RT	CR
5	75/F	PLE, SSN	3	SCLC	3	LD	Chemo	Near CR
6	60/F	SSN	7	SCLC	8	LD	Chemo, RT	PR
7	75/F	SSN, PCD	1	SCLC	8	LD	Chemo, RT ^b^	Unknown
8	73/M	SSN, PCD	1	SCLC	4	LD	Chemo, RT	CR
9	64/F	SSN	12	SCLC	5	ED	Chemo ^c^	PR
10	61/F	PLE	0.2	SCLC	0.5	ED	Chemo, RT	PR
11	52/F	SSN	5	SCLC	5	LD	Chemo, RT	n.e.
12	76/F	MN	5	SCLC	6	LD	Chemo, RT	n.e.
13	65/F	PEM	0.3	SCLC	0.5	ED	Chemo	n.e.
14	72/F	SSN, MN	3	No	n.a.	n.a.	n.a.	n.a.
15	79/M	SSN, AN	36	No	n.a.	n.a.	n.a.	n.a.
16	80/F	PCD	11	No	n.a.	n.a.	n.a.	n.a.
17	72/F	SSN, AN	11	No	n.a.	n.a.	n.a.	n.a.
18	63/F	SSN	9	No	n.a.	n.a.	n.a.	n.a.
19	72/F	PLE, SSN	4	No	n.a.	n.a.	n.a.	n.a.
20	76/M	PCD	2	No	n.a.	n.a.	n.a.	n.a.

PNS: paraneoplastic neurological syndrome; F: female; M: male; PLE: paraneoplastic limbic encephalitis; SSN: subacute sensory neuronopathy; AN: autonomic neuropathy; PCD: paraneoplastic cerebellar degeneration; MN: motor neuronopathy; PEM: paraneoplastic encephalomyelitis; SCLC: small-cell lung cancer; ED: extensive disease; LD: limited disease; Chemo: chemotherapy; RT: radiotherapy; PR: partial response; CR: complete response; n.a.: not applicable; n.e.: not evaluable.

^a-c^patients receiving treatment outside the study period; Time to start chemotherapy: –800 days (a), +155 days (b), and –217 days (c).

Nine patients received a short course of immunotherapy (iv methylprednisolone (ivMP) or iv immunoglobulins (ivIg)), with a median of 28 days (IQR 18–64) prior to the start of natalizumab treatment. Eight out of nine patients subsequently progressed prior to study inclusion. All patients received a structured tumor workup, including FDG-PET/CT imaging. Thirteen patients had a tumor, all SCLC, diagnosed median 5 months from onset of Hu-PNS (IQR 3–6, range 0.5–8). Ten patients received standard chemotherapy (a platinum-based drug plus etoposide) for SCLC concomitant with natalizumab. The remaining three patients received chemotherapy outside the study period, two patients before (–800 and –217 days) and one patient after (+155 days) the study. None of the patients received PD-(L)1checkpoint inhibitors for SCLC (extended disease), since this was not standard care in The Netherlands during the study period. No adverse effects were observed due to the combination of chemotherapy and natalizumab treatments. Thirteen patients (65%) completed a total of three natalizumab cycles, and the remaining seven patients received one or two cycles (Table 2). Reasons for discontinuing study treatment included: four patients died, two patients experienced too high a burden continuing the visits to our clinic, and one patient developed uncontrollable anxiety for the study treatment. There were no serious adverse events (SAE) related to natalizumab treatment, and none of the patients withdrew because of natalizumab toxicity.

**Table 2.** Natalizumab Treatment and Outcome

No.	No. of Natalizumab Cycles	Reason Early Treatment Termination	mRS Start	mRS 12 Weeks	Functional Outcome	Positive Treatment Response	EFIT Start	EFIT 12 Weeks	Neurological Outcome	Onset to Last FU (months)	Dead/Alive at Last FU
1	3	n.a.	4	3	**Improved**	Yes	2	2	Stable	16	Alive
2	3	n.a.	3	3	**Stable**	Yes	2	2	Stable	75	Alive
3	3	n.a.	3	3	**Stable**	Yes	2	2	Stable	28	Alive
4	2	Study burden	5	5	**Stable**	No	3	2 ^b^	Improved	22	Alive
5	3	n.a.	4	4	**Stable**	No	3	3	Stable	20	Dead
6	3	n.a.	3	3	**Stable**	Yes	1	1	Stable	33	Alive
7	3	n.a.	4	4	**Stable**	No	2	2	Stable	25	Dead
8	1	Study burden	4	5	**Worse**	No	3	-	n.a.	18	Alive
9	2	Died	3	6	**Worse**	No	1	2 ^b^	Worse	15	Dead
10	3	n.a.	3	4	**Worse ** ^a^	No	4	2	Improved ^a^	7	Dead
11	1	Died	5	6	**Worse**	No	2	-	n.a.	6	Dead
12	1	Died	5	6	**Worse**	No	3	-	n.a.	7	Dead
13	1	Died	5	6	**Worse**	No	3	4 ^b^	Worse	2	Dead
14	3	n.a.	4	3	**Improved**	Yes	3	3	Stable	23	Dead
15	3	n.a.	2	2	**Stable**	Yes	2	2	Stable	41	Alive
16	3	n.a.	2	2	**Stable**	Yes	1	1	Stable	40	Dead
17	1	Study anxiety	3	3	**Stable**	Yes	1	-	n.a.	18	Alive
18	3	n.a.	3	3	**Stable**	Yes	1	1 ^c^	Stable	15	Alive
19	3	n.a.	4	4	**Stable**	No	3	3	Stable	13	Dead
20	3	n.a.	4	4	**Stable**	No	2	3	Worse	13	Alive

mRS: modified Rankin Scale; EFIT: Edinburgh Functional Impairment Tests; FU: follow-up; n.a.: not applicable.

^a^Functional outcome was worse due to tumor progression while the neurological outcome remained improved.

^b^EFIT score 4 weeks after baseline.

^c^EFIT score 8 weeks after baseline.

**Figure 2.**
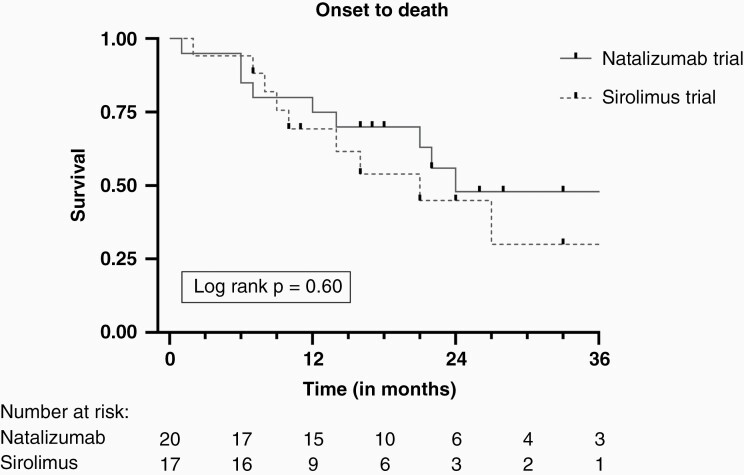
Kaplan-Meier estimates of survival for patients treated with natalizumab or sirolimus. Kaplan-Meier estimates of survival in Hu-PNS patients. Patients were divided into two groups based on natalizumab or sirolimus treatment. Survival in the natalizumab trial is depicted with the continuous line and survival in the sirolimus trial is depicted with the dashed line.

In total, ten patients had died at the last follow-up, and the cause of death was PNS in four patients, in another three patients it was due to the tumor, and three patients requested euthanasia (Supplementary Table 2). Patients were followed from onset for a total of 19 months (IQR 13–27) and median overall survival was 13 months (Figure 2).

### Outcome Measures

Two patients (No. 1 and 14) reached the primary endpoint as they had a decrease of one point in mRS score compared to baseline (10%, Table 2). They had stable or improved scores on the secondary outcome measures. Both patients had a combined sensorimotor neuronopathy (with accompanying limbic encephalitis symptoms in one). During treatment muscle strength improved and both regained the ability to walk without help. The mRS remained stable in twelve patients (60%), while six patients (30%) had further functional deterioration (Supplementary Figure 1). mRS scores per patient did not differ at timepoints 12 and 20 weeks. Nine patients (45%) were classified as treatment responders according to the Keime-Guibert criteria.^18^ Both patients who improved by mRS had central nervous system involvement, while 9/18 patients who did not improve had only peripheral nervous system involvement (*P* = .48). Measuring a positive treatment response, 6/9 had only peripheral involvement, while 8/11 non-responders had central nervous system or combined involvement (*P* = .17).

At 12 weeks, the secondary endpoints were evaluable in thirteen patients (four patients died and three had other reasons for discontinuing study visits). Two patients improved on the EFIT scale, of whom one patient (No. 10) remained stable on the mRS scale until her functional status deteriorated due to tumor progression, and the other patient (No. 4) had stable mRS scores during the whole study period. Six out of sixteen patients improved on the BI (≥5 points), of whom five were treatment responders.

The patients classified as treatment responders had significantly better baseline mRS, EFIT, and BI scores than the non-responders (Table 3). In addition, the time from onset of symptoms to Hu-PNS diagnosis was significantly longer in responders (9 vs 2 months, *P* = .008), probably reflecting the milder disease. First-line immunotherapy (ivMP or ivIg) was not associated with treatment response. Within the group of responders, fewer underlying tumors were detected and of the underlying tumors, more achieved complete remission. However, these changes were not significant. Patients with a tumor had a lower age at diagnosis and a worse mRS score at the end of the study, while all other characteristics did not differ significantly (Supplementary Table 3, and Supplementary Figure 2).

**Table 3.** Treatment Response According to Keime-Guibert Criteria^a^

	Treatment Response (*n* = 9)	No Treatment Response (*n* = 11)	*P* value
Gender, female	8 (89%)	9 (82%)	1.00
Age at onset (median, IQR, range)	66 (61–75, 57–80)	72 (61–75, 52–76)	.94
Onset to diagnosis, months (median, IQR, range)	9 (4–23, 3–62)	2 (1–5, 0.2–12)	.008
PNS syndrome, only peripheral nervous system involvement	6/9 (67%)	3/11 (27%)	.17
Tumor			
Tumor (all SCLC)	4 (44%)	9 (82%)	.16
Onset to tumor, months	6.0 (1.1)	4.0 (2.6)	.17
Tumor stage—ED	1 (25%)	3 (33%)	1.00
Tumor response—CR	3/4 (75%)	2/6 (33%)	.52
Tumor response—PR and near CR	1/4 (25%)	3/6 (50%)	.57
Ancillary testing			
Serum Hu titer, start (median, IQR, range)	1600 (1200–3200, 400–6400)	3200 (800–6400, 400–>10000)	.36
Serum Hu titer, 12 weeks (*n* = 11) (median, IQR, range)	800 (400–1600, 0–6400)	3200 (2000–3200, 1600–3200)	.082
CSF Hu titer, start (median, IQR, range)	24 (4–104, 0–512)	64 (26–160, 2–256)	.24
CSF Hu titer, 12 weeks (*n* = 9) (median, IQR, range)	2 (0–48, 0–64)	34 (4–112, 4–128)	.17
WBC (mean, SD)	4.2 (2.3)	9.1 (7.6)	.067
WBC elevated	2 (22%)	6 (55%)	.20
Total protein elevated	4 (44%)	7 (64%)	.65
IgG index elevated	1 (11%)	1 (9%)	1.00
Oligoclonal bands present	3/6 (50%)	3/4 (75%)	.57
Treatment			
Immunotherapy before trial	2 (22%)	7 (64%)	.092
No. Natalizumab cycles (median, IQR, range)	3 (3–3, 1–3)	2 (1–3, 1–3)	.065
Outcome			
mRS, baseline(median, IQR, range)	3 (2–3, 2–4)	4 (4–5, 3–5)	.005
mRS, follow-up (n=20)(median, IQR, range)	3 (2–3, 2–3)	4 (4–6, 4–6)	<.0001
EFIT, baseline(median, IQR, range)	2 (1–2, 1–3)	3 (2-3, 1-4)	.015
EFIT, follow-up (n=16)(median, IQR, range)	2 (1–2, 1–3)	3 (2-3, 2-4)	.036
BI, baseline(median, IQR, range)	95 (57–97, 20–100)	40 (25–70, 5–885)	.012
BI, follow-up (n=16)(median, IQR, range)	90 (81–98, 55–100)	40 (12–53, 10–75)	.001
Follow-up			
mRS lastfollow-up(median, IQR, range)	5 (3–6, 3–6)	6 (5–6, 3–6)	.33
Dead at last follow-up	2 (22%)	8 (73%)	.070
Onset to death, months (median, IQR, range)	30 (22–n.a., 22–39)	9 (6–19, 1–24)	.07

IQR: interquartile range; PNS: paraneoplastic neurological syndrome; SSN: subacute sensory neuronopathy; SCLC: small-cell lung cancer; ED: extensive disease; CR: complete response; PR: partial response; WBC: white blood cell count; SD: standard deviation; mRS: modified Rankin Scale; EFIT: Edinburgh Functional Impairment Tests; BI: Barthel Index.

Data are *n* (%), *n/n* (%), median (SD), or median (IQR, range).

^a^A positive treatment response was defined as improvement or stabilization in patients with an mRS score ≤3, and improvement from mRS ≥4 to mRS ≤3.^18^

### Ancillary Testing

In all patients, CSF was collected prior to the start of treatment. An elevated white blood cell count was present in eight patients (40%; maximum 26 WBC/uL), 11 (55%) had elevated total protein levels, two (10%) had increased IgG index, and 6/10 patients had oligoclonal bands. All these parameters were normal in five patients (25%). Nine patients consented to a second CSF evaluation after treatment which showed neither differences in WBC nor in total protein elevation (both *P* = 1.00). Hu-Ab median CSF titer at baseline was 32 (IQR 14–128) and 4 (IQR 1–64) after treatment (*P* = .67). In serum, median titer was 3200 before (IQR 1000–3200) and 1600 (IQR 800–3200) after treatment (*P* = .37; Supplementary Table 4). Hu-ab titers neither correlated with baseline mRS, nor with mRS change during follow-up.

IHC showed in all 20 patients’ sera and CSF the typical Hu-staining pattern, and 18 were negative for additional antibodies. One patient’s CSF showed a strong positive neuropil staining pattern, and antibody binding to membrane-bound proteins was confirmed using live hippocampal neurons. Results for anti-GABA_B_R, anti-AMPAR, anti-VGKC, anti-CASPR2, anti-LGI1, anti-NMDAR, anti-GAD, anti-DPPX, anti-IgLON5, anti-VGCC, anti-CNTN1, anti-NF155 antibodies, all returned negative. This patient (No. 13) had encephalomyelitis, SCLC, and high baseline Hu-Ab titers (serum 1:3200; CSF 1:64). Prior to diagnosis, she received ivIg without improvement. The patient died after one cycle of natalizumab. The CSF of another patient (No. 6) showed an atypical staining pattern on IHC, suitable with AQP4. This was confirmed by a CBA in serum. This patient presented with SSN, had SCLC and high Hu-Ab titers (serum 1:3200; CSF 1:16). SSN remained stable during the study period. Six months after natalizumab treatment, she developed optic neuritis attributed to the anti-AQP4 antibodies.

### Comparison with Treatment Response from Historical Hu-PNS Patients

As we compared our data to the sirolimus trial^21^ no difference in functional outcome was observed, 10% vs 6% showed improvement in mRS (*P* = .87; Supplementary Table 1). In addition, treatment response was similar between the two cohort (45% vs 41% responders, *P* = .82), as was neurological outcome (*P* = .53). The natalizumab cohort was comparable to the sirolimus cohort, but for a longer duration to tumor diagnosis (median 5 vs 2 months, *P* = .036). Baseline mRS appeared higher in the natalizumab cohort (median 4 vs 3, *P* = .18), without reaching statistical significance.

## Discussion

In this prospective open-label trial administering natalizumab in patients with Hu-PNS, we show that objective functional improvement is rare and achieved in 10%, while a stable situation was obtained in another 60%. Ascertained by the Keime-Guibert criteria, treatment response was classified as positive in 45%. As all patients had progressive neurological symptoms in the four weeks prior to inclusion, the high percentage of functional improvement and stabilization (70% together) suggests some efficacy of natalizumab. However, due to the non-randomized design of our study, it cannot be excluded that stabilization reflected the natural course of the disease as Hu-PNS ultimately reaches a plateau phase.^1–3^

Published studies of immunosuppression or immunomodulation in Hu-PNS using the same mRS based outcome criteria evaluated various treatments, including plasma exchange, ivMP, cyclophosphamide, ivIg, rituximab, and human chorionic gonadotropin.^18,23–30^ These studies found similar rates of objective functional improvement (0–40%, pooled 11%) and stabilization (20–71%, pooled 49%). Also, the treatment response was classified as positive in 0–65% (pooled 42%) of patients in these studies, similar to the positive response we found in 45%.^18,23–30^ In our institution, an earlier trial in patients with Hu-PNS was conducted by De Jongste et al.^21^ treating patients with sirolimus (activated T cell suppressor). We used this cohort as a control group after showing that there were no relevant differences between the two cohorts. Treatment with sirolimus or natalizumab showed similar results in all outcome measures.

Previous studies in Hu-PNS found that in patients with a tumor, the functional outcome is better with antitumor treatment.^1,22,31^ In our study, the outcome in the three patients with a tumor not receiving concomitant antitumor treatment was similar to the ten patients receiving concomitant antitumor therapy. As previously observed, we saw a trend in better functional outcome in patients without a tumor compared to patients with a tumor.^29^ Five of nine patients with a positive outcome received only natalizumab without concomitant antitumor treatment indicating that immunosuppression may ameliorate the disease course.^22^ In patients receiving both chemotherapy and immunosuppressive or immunomodulatory therapy, it is unclear whether the immunotherapy has an additional effect.

As functional improvement is rare with the currently available treatment modalities, stabilization of the patient seems the most realistic treatment goal. Because of the rapidly progressive course of the disease, early diagnosis with the patient in a better condition is warranted. Indeed, moderate disability (mRS ≤ 3) at start of treatment associates with a more favorable outcome.^29^ Unfortunately, the median time from symptom onset to diagnosis was 5 months, which has not improved over the last 20 years.^1,26,27,29^ By this time, most patients already have severe symptoms, probably reflecting extensive and irreversible neuronal loss.

In Hu-PNS, patients can harbor other neuronal autoantibodies including those recognizing surface antigens.^2,32^ In these patients the neurological syndrome may be caused by the cell-surface antibodies while the Hu-Ab may be biomarkers of an underlying SCLC (15% of SCLC harbor Hu-Ab, most without PNS).^33^ As their treatment strategies, response, and outcome may be different, we screened for cell-surface antibodies. We identified a second antibody in two patients: one patient with a currently unidentified antibody and one with anti-AQP4 antibodies, a rare accompaniment. In both patients, the clinical presentation and disease course were typical of Hu-PNS. The second patient developed optic neuritis six months after natalizumab treatment, most likely related to anti-AQP4 antibodies. We found no GABA_B_R antibodies, the most frequently described co-occurrence with Hu-Ab.^34,35^

Limitations of our study are the small sample size and the open-label non-randomized design. A marginal positive effect of natalizumab cannot be excluded as the trial was not powered to detect a difference in effect <25% compared to historic studies. These limitations are directly related to the low incidence of Hu-PNS and the difficulty to accrue patients who are still in the progressive phase of the disease. Due to the severity of the disease, a high percentage of trial candidates were unwilling to participate in a trial outside their own region. This could have been a source for selection bias. However, our cohort still consisted of patients with a high mRS at baseline, similar to other studies in this field. Seven patients chose not to complete all three cycles of natalizumab. Some secondary or exploratory outcome measures could not be collected in these patients. However, as the mRS scores were always available, this did not change the primary outcome of our study. In our trial, almost half of the patients had received a form of first-line immunotherapy (ivMP or ivIg or both) in the referral hospital before the start of natalizumab treatment. As all but one of these patients had evident neurological progression prior to inclusion in the study, first-line immunotherapy is unlikely to have influenced the results. Finally, many of our patients had involvement of dorsal root ganglia (SSN) and there are very few data on the effect of natalizumab on the traffic of T cells into dorsal root ganglia.^36^ Natalizumab may theoretically be less effective in blocking T cell traffic into dorsal root ganglia than traffic into the central nervous system. However, we did in our study not observe better efficacy of natalizumab in patients with central or combined central and peripheral nervous system involvement than in patients with involvement of peripheral nervous system only.

To conclude, natalizumab may ameliorate the disease course in Hu-PNS. However, the efficacy of natalizumab seems not superior to other immunosuppressive and immunomodulatory treatment strategies. Rapid diagnosis of Hu-PNS followed by tumor identification and treatment are essential to stabilize the patient when still ambulatory. In patients without a tumor, or not receiving antitumor treatment for another reason, immunosuppressive or immunomodulatory therapies should be seriously considered. Until now, there is no preferred choice in the kind of immunotherapy. Better, more effective treatments are clearly still needed.

## Supplementary Material

vdab145_suppl_Supplementary_Data

## References

[1] Graus F , Keime-Guibert F, Reñe R, et al. Anti-Hu-associated paraneoplastic encephalomyelitis: analysis of 200 patients. Brain. 2001;124(Pt 6):1138–1148. 11353730 10.1093/brain/124.6.1138

[2] Sillevis Smitt P , Grefkens J, de Leeuw B, et al. Survival and outcome in 73 anti-Hu positive patients with paraneoplastic encephalomyelitis/sensory neuronopathy. J Neurol. 2002;249(6):745–753. 12111309 10.1007/s00415-002-0706-4

[3] Dalmau J , Graus F, Rosenblum MK, Posner JB. Anti-Hu–associated paraneoplastic encephalomyelitis/sensory neuronopathy. A clinical study of 71 patients. Medicine (Baltimore). 1992;71(2):59–72. 1312211 10.1097/00005792-199203000-00001

[4] Lucchinetti CF , Kimmel DW, Lennon VA. Paraneoplastic and oncologic profiles of patients seropositive for type 1 antineuronal nuclear autoantibodies. Neurology. 1998;50(3):652–657. 9521251 10.1212/wnl.50.3.652

[5] Darnell RB , Posner JB. Paraneoplastic syndromes involving the nervous system. N Engl J Med. 2003;349(16):1543–1554. 14561798 10.1056/NEJMra023009

[6] Sillevis Smitt PA , Manley GT, Posner JB. Immunization with the paraneoplastic encephalomyelitis antigen HuD does not cause neurologic disease in mice. Neurology. 1995;45(10):1873–1878. 7477985 10.1212/wnl.45.10.1873

[7] Bien CG , Vincent A, Barnett MH, et al. Immunopathology of autoantibody-associated encephalitides: clues for pathogenesis. Brain. 2012;135(Pt 5):1622–1638. 22539258 10.1093/brain/aws082

[8] Bernal F , Graus F, Pifarré A, Saiz A, Benyahia B, Ribalta T. Immunohistochemical analysis of anti-Hu-associated paraneoplastic encephalomyelitis. Acta Neuropathol. 2002;103(5):509–515. 11935268 10.1007/s00401-001-0498-0

[9] Bielekova B , Becker BL. Monoclonal antibodies in MS: mechanisms of action. Neurology. 2010;74(Suppl 1):S31–S40. 20038761 10.1212/WNL.0b013e3181c97ed3 PMC11335136

[10] del Pilar Martin M , Cravens PD, Winger R, et al. Decrease in the numbers of dendritic cells and CD4+ T cells in cerebral perivascular spaces due to natalizumab. Arch Neurol. 2008;65(12):1596–1603. 18852339 10.1001/archneur.65.12.noc80051

[11] de Andrés C , Teijeiro R, Alonso B, et al. Long-term decrease in VLA-4 expression and functional impairment of dendritic cells during natalizumab therapy in patients with multiple sclerosis. PLoS One. 2012;7(4):e34103. 22496780 10.1371/journal.pone.0034103 PMC3319565

[12] van Swieten JC , Koudstaal PJ, Visser MC, Schouten HJ, van Gijn J. Interobserver agreement for the assessment of handicap in stroke patients. Stroke. 1988;19(5):604–607. 3363593 10.1161/01.str.19.5.604

[13] Ances BM , Vitaliani R, Taylor RA, et al. Treatment-responsive limbic encephalitis identified by neuropil antibodies: MRI and PET correlates. Brain. 2005;128(Pt 8):1764–1777. 15888538 10.1093/brain/awh526 PMC1939694

[14] Gresa-Arribas N , Titulaer MJ, Torrents A, et al. Antibody titres at diagnosis and during follow-up of anti-NMDA receptor encephalitis: a retrospective study. Lancet Neurol. 2014;13(2):167–177. 24360484 10.1016/S1474-4422(13)70282-5 PMC4006368

[15] European Medicines Agency: Summary of Product Characteristics Tysabri; [Available from: https://www.ema.europa.eu/en/documents/product-information/tysabri-epar-product-information_en.pdf. Accessed May 2021.

[16] Chang SM , Reynolds SL, Butowski N, et al. GNOSIS: guidelines for neuro-oncology: standards for investigational studies-reporting of phase 1 and phase 2 clinical trials. Neuro Oncol. 2005;7(4):425–434. 16212807 10.1215/S1152851705000554 PMC1871726

[17] Bruno A , Shah N, Lin C, et al. Improving modified Rankin Scale assessment with a simplified questionnaire. Stroke. 2010;41(5):1048–1050. 20224060 10.1161/STROKEAHA.109.571562

[18] Keime-Guibert F , Graus F, Fleury A, et al. Treatment of paraneoplastic neurological syndromes with antineuronal antibodies (Anti-Hu, anti-Yo) with a combination of immunoglobulins, cyclophosphamide, and methylprednisolone. J Neurol Neurosurg Psychiatry. 2000;68(4):479–482. 10727484 10.1136/jnnp.68.4.479 PMC1736897

[19] Clyde Z , Chataway SJ, Signorini D, Gregor A, Grant R. Significant change in tests of neurological impairment in patients with brain tumours. J Neurooncol. 1998;39(1):81–90. 9760073 10.1023/a:1005950003774

[20] Mahoney FI , Barthel DW. Functional evaluation: the Barthel Index. MD State Med J. 1965;14:61–65. 14258950

[21] de Jongste AH , van Gelder T, Bromberg JE, et al. A prospective open-label study of sirolimus for the treatment of anti-Hu associated paraneoplastic neurological syndromes. Neuro Oncol. 2015;17(1):145–150. 24994790 10.1093/neuonc/nou126 PMC4483045

[22] Keime-Guibert F , Graus F, Broët P, et al. Clinical outcome of patients with anti-Hu-associated encephalomyelitis after treatment of the tumor. Neurology. 1999;53(8):1719–1723. 10563618 10.1212/wnl.53.8.1719

[23] Graus F , Vega F, Delattre JY, et al. Plasmapheresis and antineoplastic treatment in CNS paraneoplastic syndromes with antineuronal autoantibodies. Neurology. 1992;42(3 Pt 1):536–540. 1312683 10.1212/wnl.42.3.536

[24] Uchuya M , Graus F, Vega F, Reñé R, Delattre JY. Intravenous immunoglobulin treatment in paraneoplastic neurological syndromes with antineuronal autoantibodies. J Neurol Neurosurg Psychiatry. 1996;60(4):388–392. 8774401 10.1136/jnnp.60.4.388 PMC1073889

[25] Vernino S , O’Neill BP, Marks RS, O’Fallon JR, Kimmel DW. Immunomodulatory treatment trial for paraneoplastic neurological disorders. Neuro Oncol. 2004;6(1):55–62. 14769141 10.1215/S1152851703000395 PMC1871966

[26] Shams’ili S , de Beukelaar J, Gratama JW, et al. An uncontrolled trial of rituximab for antibody associated paraneoplastic neurological syndromes. J Neurol. 2006;253(1):16–20. 16444604 10.1007/s00415-005-0882-0

[27] van Broekhoven F , de Graaf MT, Bromberg JE, et al. Human chorionic gonadotropin treatment of anti-Hu-associated paraneoplastic neurological syndromes. J Neurol Neurosurg Psychiatry. 2010;81(12):1341–1344. 20667866 10.1136/jnnp.2009.177865

[28] Berzero G , Karantoni E, Dehais C, et al. Early intravenous immunoglobulin treatment in paraneoplastic neurological syndromes with onconeural antibodies. J Neurol Neurosurg Psychiatry. 2018;89(7):789–792. 29084869 10.1136/jnnp-2017-316904 PMC6031268

[29] de Jongste AH , van Rosmalen J, Gratama JW, Sillevis Smitt P. Current and future approaches for treatment of paraneoplastic neurological syndromes with well-characterized onconeural antibodies. Exp Opin Orphan Drugs. 2014;5(2):483–496.

[30] Berzero G , Psimaras D. Neurological paraneoplastic syndromes: an update. Curr Opin Oncol. 2018;30(6):359–367. 30124520 10.1097/CCO.0000000000000479

[31] Vedeler CA , Antoine JC, Giometto B, et al.; Paraneoplastic Neurological Syndrome Euronetwork. Management of paraneoplastic neurological syndromes: report of an EFNS Task Force. Eur J Neurol. 2006;13(7):682–690. 16834698 10.1111/j.1468-1331.2006.01266.x

[32] Pittock SJ , Kryzer TJ, Lennon VA. Paraneoplastic antibodies coexist and predict cancer, not neurological syndrome. Ann Neurol. 2004;56(5):715–719. 15468074 10.1002/ana.20269

[33] Titulaer MJ , Klooster R, Potman M, et al. SOX antibodies in small-cell lung cancer and Lambert-Eaton myasthenic syndrome: frequency and relation with survival. J Clin Oncol. 2009;27(26):4260–4267. 19667272 10.1200/JCO.2008.20.6169

[34] Höftberger R , Titulaer MJ, Sabater L, et al. Encephalitis and GABAB receptor antibodies: novel findings in a new case series of 20 patients. Neurology. 2013;81(17):1500–1506. 24068784 10.1212/WNL.0b013e3182a9585f PMC3888170

[35] van Coevorden-Hameete MH , de Bruijn MAAM, de Graaff E, et al. The expanded clinical spectrum of anti-GABABR encephalitis and added value of KCTD16 autoantibodies. Brain. 2019;142(6):1631–1643. 31009048 10.1093/brain/awz094 PMC6536844

[36] Lakritz JR , Thibault DM, Robinson JA, et al. α4-Integrin antibody treatment blocks monocyte/macrophage traffic to, vascular cell adhesion molecule-1 expression in, and pathology of the dorsal root ganglia in an SIV macaque model of HIV-peripheral neuropathy. Am J Pathol. 2016;186(7):1754–1761. 27157989 10.1016/j.ajpath.2016.03.007 PMC4929389

