# Supplementary material for: Phase II trial of natalizumab for the treatment of anti-Hu associated paraneoplastic neurological syndromes
Source: Neurooncol Adv. 2021 Sep 28;3(1):vdab145. doi: 10.1093/noajnl/vdab145 (PMC8528262; doi:10.1093/noajnl/vdab145)
Supplement: vdab145_suppl_Supplementary_Data [file vdab145_suppl_supplementary_data.docx]

**Supplementary Figure 1. mRS scores during the total study period of 20 weeks.**

mRS scores per patient during the study period of 20 weeks. Patients were divided in two groups based on tumor presence. The two thick lines represents the two patients who showed improvement in mRS score.

**Supplementary Figure 2. Kaplan-Meier estimates of survival for patients with and without a tumor.**

Kaplan-Meier estimates of survival in Hu-PNS patients. Patients were divided into two groups based on tumor presence. Tumor presence is depicted with the continuous line and no tumor is depicted with the dashed line.

| **Supplementary Table 1. Cohort characteristics and comparison between patients treated with natalizumab and a prior study with sirolimus treatment ^21^** | | | | |
| --- | --- | --- | --- | --- |
|  | | **Natalizumab trial (n=20)** | **Sirolimus trial (n=17)** | **p value** |
| Gender, female | | 17 (85%) | 11 (65%) | 0.25 |
| Age at onset (mean, SD) | | 67.8 (8.4) | 65.2 (8.2) | 0.35 |
| Onset to diagnosis, months (median, IQR, range) | | 5 (2-11, 0.2-62) | 4 (1-6, 0-11) | 0.12 |
| PNS syndrome | |  |  | 1.00^b^ |
|  | SSN | 6 (30%) | 6 (35%) |  |
|  | SSN with other peripheral nervous syndrome | 3 (15%) | 1 (6%) |  |
|  | SSN with other central nervous syndrome | 6 (30%) | 2 (12%) |  |
|  | PLE | 1 (5%) | 2 (12%) |  |
|  | MN | 1 (5%) | 1 (6%) |  |
|  | PCD | 2 (10%) | 3 (18%) |  |
|  | PEM | 1 (5%) | 1 (6%) |  |
| **Tumor** | | | | |
| Tumor | | 13 (65%); all SCLC | 15 (88%); 3 no biopsy; 10 SCLC | 0.14 |
| Onset to tumor diagnosis, months (median, IQR, range) | | 5 (3-6, 0.5-8) | 2 (-4-5, -312-8) | **0.036** |
| Tumor stage | |  |  | 0.35 |
|  | LD | 9/13 (69%) | 11/13 (85%) |  |
|  | ED | 4/13 (31%) | 2/13 (15%) |  |
| Chemotherapy | | 13/13 (100%) | 10/12 (83%) | 0.22 |
| Tumor response | |  |  | 0.65 |
|  | CR | 5/9 (56%) | 7/10 (70%) |  |
|  | near CR | 1/9 (11%) | 0 |  |
|  | PR | 3/9 (33%) | 1/10 (10%) |  |
|  | PD | 0 | 2/10 (20%) |  |
|  | N.E. | 3 | 0 |  |
| **Ancillary testing** | | | | |
| Serum Hu titer, baseline (median, IQR, range) | | 3200 (1000-3200, 400->10000) | 3200 (1200-6400, 400-12800) | 0.39 |
| Serum Hu titer, 12 weeks (median, IQR, range) | | 1600 (800-3200, 0-6400); n=11 | 1600 (800-6400, 800-6400); n=11 | 0.52 |
| CSF Hu titer, baseline (median, IQR, range) | | 32 (14-128, neg-512); n=18 | 32 (8-256, 8-2048); n=15 | 0.76 |
| CSF Hu titer, 12 weeks (median, IQR, range) | | 4 (1-64, 0-128); n=9 | 48 (10-224, 0-1024); n=12 | 0.11 |
| Routine CSF normal | | 5 (25%) | 5 (29%) |  |
|  | WBC (median, IQR, range) | 4 (3-9, 1-26) | 5 (2-11, 1-49) | 0.87 |
|  | WBC elevated | 8 (40%) | 8 (47%) | 0.67 |
|  | Total protein elevated | 11 (55%) | 7 (41%) | 0.40 |
|  | IgG index elevated | 2 (10%) | 3 (18%) | 0.64 |
|  | Oligoclonal bands | 6/10 (60%) | 9/13 (69%) | 0.69 |
| **Treatment** | | | | |
| Immunotherapy before trial | | 9 (45%) | 3 (18%) | 0.09 |
|  | ivMP | 2 | 3 |  |
|  | ivIg | 4 | 3 |  |
|  | ivMP+ivIg | 3 | 0 |  |
|  | Immunotherapy to start trial, days (median, IQR, range) | 28 (18-64, 8-96) | n.e. |  |
| No. Natalizumab cycles (median, IQR, range) | | 3 (1-3, 1-3) | n.a. |  |
| **Outcome** | | | | |
| Positive treatment response ^a^ | | 9 (45%) | 7 (41%) | 0.82 |
| Functional outcome (mRS) | |  |  | 0.87 |
|  | Improved | 2 (10%) | 1 (6%) |  |
|  | Stable | 12 (60%) | 10 (59%) |  |
|  | Worse | 6 (30%) | 6 (35%) |  |
|  | mRS, baseline (median, IQR, range) | 4 (3-4, 2-5) | 3 (3-4, 2-5) | 0.18 |
|  | mRS, follow-up (median, IQR, range) | 4 (3-5, 2-6) | 4 (3-4, 1-6) | 0.63 |
| Neurological outcome (EFIT) | |  |  | 0.53 |
|  | Improved | 2 (13%) | 2/14 (14%) |  |
|  | Stable | 11 (69%) | 7/14 (50%) |  |
|  | Worse | 3 (20%) | 5/14 (36%) |  |
|  | EFIT, baseline (median, IQR, range) | 2 (1-3, 1-4) | 2 (2-2, 0-4) | 0.78 |
|  | EFIT, follow-up (median, IQR, range) | 2 (2-3, 1-4) | 2 (2-3, 1-4) |  |
| BI, baseline (median, IQR, range) | | 65 (31-93, 5-100) | 85 (47-100, 15-100) | 0.17 |
| BI, follow-up (median, IQR, range) | | 65 (37-90, 10-100) | n.e. |  |
| **Follow-up** | | | | |
| Follow-up from onset, months (median, IQR, range) | | 19 (13-27, 2-75) | 14 (8-22, 2-43) | 0.24 |
| mRS at last follow-up (median, IQR, range) | | 5 (3-6, 1-6) | n.e. |  |
| Dead during study | | 4 (20%) | n.e. |  |
| Dead, total at last follow-up | | 10 (50%) | 9 (53%) | 0.86 |
| Onset to death, months (mean, SD) | | 15 (11.4) | 12 (7.6) | 0.49 |
| Cause of death | |  |  | 0.21 |
|  | PNS | 4/10 (40%) | 7/9 (78%) |  |
|  | Tumor | 3/10 (30%) | 2/9 (22%) |  |
|  | Euthanasia | 3/10 (30%) | 0 |  |

Abbreviations: SD = standard deviation, IQR = interquartile range, PNS = paraneoplastic neurological syndrome, SSN = subacute sensory neuronopathy, PLE = paraneoplastic limbic encephalitis, PCD = paraneoplastic cerebellar degeneration, MN = motor neuronopathy, PEM = paraneoplastic encephalomyelitis, SCLC = small-cell lung cancer, LD = limited disease, ED = extensive disease, CR = complete response, PR = partial response, PD = progressive disease, n.e. = not evaluable, WBC = white blood cell count, ivMP = intravenous methylprednisolone, ivIg = intravenous immunoglobulins, mRS = modified Rankin Scale, EFIT = Edinburgh Functional Impairment Tests, BI = Barthel Index.

Data are n (%), n/n (%), median (SD) or median (IQR, range).

^a^ A positive treatment response was defined as improvement or stabilization in patients with an mRS score ≤3, and improvement from mRS ≥4 to mRS ≤3.^18^

^b^ Patients with only peripheral nervous system involvement compared with patients who had only central or combined peripheral and central nervous system involvement

| **Supplementary Table 2. Characteristics of the patients who requested euthanasia.** | |
| --- | --- |
| No. 9 | Progressive sensory neuronopathy with hand function and walking difficulties.  Seven months before start of the trial she was diagnosed with SCLC, extensive disease, and treated with chemotherapy with partial response.  Four weeks after start of the trial there was neurological deterioration with cerebellar ataxia, hemiparesis and inability to walk. Imaging showed multiple (>15) brain metastases.  Due to further neurological decline in combination with the poor prognosis, the patient withdrew from the trial. |
| No. 11 | Progressive neurological dysfunction involving dysphagia resulting in tube feeding, inability to walk or sit due to severe sensory ataxia. Totally dependent.  No abnormalities on extensive MRI imaging of brain and spinal cord  No response on earlier ivIg before trial. Received one cycle of chemotherapy for SCLC, limited disease.  After start of the trial she was diagnosed with SCLC and because of her extensive disabilities in combination with the poor prognosis the patient requested no further treatment and withdrew from the trial. |
| No. 13 | Fast neurological deterioration with prominent loss of muscle strength in neck and arms, eye movement disorder, dysphagia, and respiratory failure. Bedridden. No behavior problems or cognitive decline.  No abnormalities on MRI imaging of brain, spinal cord and plexus brachialis.  ICU admittance due to progressive respiratory failure and need for ventilator (2,5 weeks).  No response on earlier ivIg before trial. Received one cycle of chemotherapy for SCLC, extensive disease.  After start of the trial she had further neurological deterioration and she was diagnosed with SCLC. The patient requested no further treatment due to her disabilities and poor prognosis and withdrew from the trial. |

| **Supplementary Table 3. Characteristics of patients with and without a tumor.** | | | | |
| --- | --- | --- | --- | --- |
|  | | **No tumor (n=7)** | **Tumor (n=13)** | **p value** |
| Gender, female | | 5 (71%) | 12 (92%) | 0.27 |
| Age at onset (mean, SD) | | 73.4 (5.7) | 64.7 (8.2) | **0.022** |
| Onset to diagnosis, months (median, IQR, range) | | 9 (3-11, 2-36) | 3 (1-6, 0.2-62) | 0.18 |
| PNS syndrome, only peripheral nervous system involvement | | 3 (43%) | 6 (46%) | 1.00 |
| **Ancillary testing** | | | | |
| Serum Hu titer, baseline (median, IQR, range) | | 1600 (400-3200, 400-3200) | 3200 (1600-6400, 400->10000) | 0.060 |
| Serum Hu titer, 12 weeks (n=11) (median, IQR, range) | | 1800 (100-5600, 0-6400) | 1600 (800-3200, 800-3200) | 0.69 |
| CSF Hu titer, baseline (median, IQR, range) | | 16 (0-48, 0-64) | 64 (24-192, 2-512) | 0.059 |
| CSF Hu titer, 12 weeks (n=9) (median, IQR, range) | | 48 (8-64, 0-64) | 4 (1-66, 0-128) | 0.54 |
| WBC (median, IQR, range) | | 4 (2-7, 2-9) | 5 (3-13, 1-26) | 0.28 |
| WBC elevated | | 2 (29%) | 6 (46%) | 0.64 |
| Total protein elevated | | 3 (43%) | 8 (61%) | 0.64 |
| IgG index elevated | | 0 | 2 (15%) | 0.52 |
| Oligoclonal bands present | | 3/5 (60%) | 3/5 (60%) | 1.0 |
| **Treatment** | | | | |
| Immunotherapy before trial | | 1 (14%) | 8 (62%) | 0.07 |
| No. Natalizumab cycles | | 3 (3-3, 1-3) | 3 (1-3, 1-3) | 0.20 |
| **Outcome** | | | | |
| Positive treatment response  ^a^ | | 5 (71%) | 4 (31%) | 0.16 |
| Functional outcome (mRS) | |  |  | 0.071 |
|  | Improved | 1 (14%) | 1 (8%) |  |
|  | Stable | 6 (86%) | 6 (46%) |  |
|  | Worse | 0 | 6 (46%) |  |
| mRS, start (median, IQR, range) | | 3 (2-4, 2-4) | 4 (3-4, 3-5) | 0.11 |
| mRS, follow-up (n=20) (median, IQR, range) | | 3 (2-4, 2-4) | 4 (3-6, 3-6) | **0.019** |
| EFIT, start (median, IQR, range) | | 2 (1-3, 1-3) | 2 (1-3, 1-3) | 0.23 |
| EFIT, follow-up (n=16) (median, IQR, range) | | 2 (2-3, 1-4) | 2 (2-2, 1-4) | 0.91 |
| BI, start (median, IQR, range) | | 80 (40-100, 20-100) | 55 (27-87, 5-95) | 0.17 |
| BI, follow-up (n=16) (median, IQR, range) | | 82 (65-92, 35-100) | 52 (17-91, 10-100) | 0.23 |
| **Follow-up** | | | | |
| Months follow-up (mean, SD) | | 25.7 (11.6) | 21.1 (18.7) | 0.56 |
| mRS last follow-up (median, IQR, range) | | 6 (3-6, 3-6) | 6 (4-6, 3-6) | 1.00 |
| Dead at last follow-up | | 3 (43%) | 7 (54%) | 1.00 |
| Onset to death, months (mean, SD) | | 24.3 (13.7) | 11.3 (8.6) | 0.098 |

Abbreviations: SD = standard deviation, IQR = interquartile range, PNS = paraneoplastic neurological syndrome, SSN = subacute sensory neuronopathy, WBC = white blood cell count, mRS = modified Rankin Scale, EFIT = Edinburgh Functional Impairment Tests, BI = Barthel Index.

Data are n (%), n/n (%), median (SD), or median (IQR, range).

^a^ A positive treatment response was defined as improvement or stabilization in patients with an mRS score ≤3, and improvement from mRS ≥4 to mRS ≤3.^18^

| **Supplementary Table 4. Anti-Hu antibody titer at baseline and after natalizumab treatment** | | | | |
| --- | --- | --- | --- | --- |
|  | **SERUM titer** | | **CSF titer** | |
| **No.** | **Baseline** | **Follow-up** | **Baseline** | **Follow-up** |
| 1 | 6400 | 1600 | 32 | n.a. |
| 2 | 1600 | 800 | 128 | 2 |
| 3 | 3200 | 1600 | 512 | n.a. |
| 4 | 6400 | n.a. | 128 | n.a. |
| 5 | 3200 | 1600 | 256 | 4 |
| 6 | 3200 | 800 | 16 | negative |
| 7 | 400 | 3200 | 256 | n.a. |
| 8 | 6400 | n.a. | 128 | n.a. |
| 9 | 10000 | n.a. | 8 | 4 |
| 10 | 3200 | 3200 | 32 | 128 |
| 11 | 1600 | n.a. | 32 | n.a. |
| 12 | 800 | n.a. | 2 | n.a. |
| 13 | 3200 | n.a. | 64 | n.a. |
| 14 | 3200 | 6400 | 32 | 64 |
| 15 | 1600 | negative | negative | negative |
| 16 | 800 | 400 | 16 | 32 |
| 17 | 400 | n.a. | negative | n.a. |
| 18 | 1600 | n.a. | n.a. | n.a. |
| 19 | 3200 | 3200 | n.a. | 64 |
| 20 | 400 | n.a. | 64 | n.a. |

Abbreviations: n.a. = not applicable.

Anti-Hu antibody median CSF titer at baseline was 32 (IQR 14-128) and 4 (IQR 1-64) after treatment at 12 weeks (p = 0.67). In serum, median titer was 3200 before (IQR 1000-3200) and 1600 (IQR 800-3200) after treatment (p = 0.37). Wilcoxon matched pairs test was used for statistical analysis.

**References**

18. Keime-Guibert F, Graus F, Fleury A, et al. Treatment of paraneoplastic neurological syndromes with antineuronal antibodies (Anti-Hu, anti-Yo) with a combination of immunoglobulins, cyclophosphamide, and methylprednisolone. J Neurol Neurosurg Psychiatry. 2000;68(4):479-482.

21. de Jongste AH, van Gelder T, Bromberg JE, et al. A prospective open-label study of sirolimus for the treatment of anti-Hu associated paraneoplastic neurological syndromes. Neuro Oncol. 2015;17(1):145-150.
